# Access to Care Among Individuals Who Experienced Medicaid Lockouts After Premium Nonpayment

**DOI:** 10.1001/jamanetworkopen.2019.14561

**Published:** 2019-11-06

**Authors:** Brendan Saloner, Laura Dague, Donna Friedsam, Kristen Voskuil, Natalia Serna Borrero, Marguerite Burns

**Affiliations:** 1 Department of Health Policy and Management, Johns Hopkins Bloomberg School of Public Health, Baltimore, Maryland; 2 Bush School of Government and Public Service, Texas A&M University, College Station; 3 University of Wisconsin, Madison

## Abstract

This survey study compares the demographic characteristics, access to care, and health status of recent Medicaid enrollees in Wisconsin with and without experience of Medicaid lockouts.

## Introduction

As of September 2019, 5 states have received federal waivers to temporarily suspend Medicaid eligibility for individuals who fail to pay required premiums.^1^ These periods, ranging from 3 to 12 months, are called *lockouts*, *noneligibility periods*, or *restrictive reenrollment periods*. Discontinuous Medicaid coverage is linked to poor access,^2,3^ but the effects of temporary, short-term lockouts are unknown. Wisconsin received a Section 1115 waiver allowing the application of premiums enforced by lockouts among adults receiving transitional medical assistance (TMA),^4^ a federally required Medicaid category that provides time-limited coverage to parents and/or caretakers when their incomes increase to exceed the Medicaid program maximum.^5^ Wisconsin did not expand Medicaid under the Patient Protection and Affordable Care Act; TMA covers some individuals who would otherwise be eligible for the expansion. This survey study compared the demographic characteristics, access to care, and health status of recent Medicaid enrollees with and without lockout experience.

## Methods

We conducted cross-sectional surveys in 2016 and 2018 among individuals who were currently or recently enrolled in TMA, stratified by lockout experience. Survey participants were informed that their participation was entirely voluntary and could not affect their program benefits. The study was a contracted program evaluation and thus was designated as not research by the University of Wisconsin-Madison institutional review board. This study followed the American Association for Public Opinion Research (AAPOR) reporting guideline.

The lockout group included individuals in at least the second month of a lockout from TMA coverage when the sample was drawn. The comparison group comprised individuals enrolled in TMA at the time the sample was drawn. Questionnaires were mailed in April 2016 and April 2018, with follow-up phone calls to nonrespondents in July of each year. Participants did not explicitly provide consent but had the option to not mail in a response or break off the interview. We pooled data from 2016 and 2018 to increase sample size; results were broadly similar between the 2 years. The sample consisted of individuals who completed the survey. We reported American Association for Public Opinion Research response rate 3 (ie, complete interviews per number eligible).

We weighted respondents in each group to account for differential nonresponse. We examined unadjusted demographic differences between the groups, then adjusted for demographic characteristics to compare self-reported access to care, health status, and insurance status in the 2 groups. We used 2-sided *t *tests to determine differences between groups and set *P* < .05 as the level for statistical significance. Analyses were conducted using Stata version 15 (StataCorp). Additional demographic characteristics of the survey sample vs sampling frame appear in eTable 1 in the Supplement, and demographic characteristics of individuals receiving TMA vs other parents or caretakers are shown in eTable 2 of the Supplement.

## Results

In 2016, the response rate was a weighted 30.2% (119 of 400) in the lockout group and a weighted 52.8% (317 of 600) in the TMA group. In 2018, it was 35.8% (59 of 157) and 44.1% (394 of 893), respectively. Pooling across years, the 178 individuals in the lockout sample were significantly less likely than the 711 individuals in the TMA group to be older than 35 years (73 [46.0%] vs 427 [61.9%]; *P* < .001) and more likely to be black non-Hispanic individuals (44 [23.7%] vs 69 [10.0%]; *P* < .001) (Table 1). A greater percentage of individuals in the lockout group than in the TMA group had a high school diploma (142 [85.2%] vs 530 [79.1%]) and did not live with a spouse (119 [66.9%] vs 463 [65.2%]) (Table 1).

**Table 1.** Demographic Characteristics of Recent Medicaid Enrollees With Lockout Experience vs Those Without Lockout Experience

Characteristic	No. (Weighted %)		*P* Value
	Individuals With Recent Lockout Experience (n = 178)^a^	Individuals With No Recent Lockout Experience (n = 711)^a^	
Male	33 (27.3)	157 (23.3)	.35
Aged >35 y	73 (46.0)	427 (61.9)	<.001
Race/Ethnicity			
White, non-Hispanic	89 (52.6)	504 (70.9)	<.001
Black, non-Hispanic	44 (23.7)	69 (10.0)	<.001
Spanish, Hispanic, or Latino	20 (10.6)	50 (6.9)	.14
Other race, not Hispanic^b^	10 (5.5)	47 (7.0)	.45
Mixed race, not Hispanic	4 (2.9)	19 (2.9)	.97
Missing	11 (4.7)	12 (2.4)	.16
≥High school diploma	142 (85.2)	530 (79.1)	.06
Household annual income <$30 000	113 (64.2)	388 (58.8)	.23
Household composition			
Lives alone	13 (7.1)	44 (6.9)	.96
Lives with spouse	38 (24.0)	210 (30.7)	.045
Lives with others	106 (63.9)	419 (59.9)	.81
Missing	21 (5.0)	38 (2.5)	.17
≥2 Household members aged <19 y	66 (37.3)	275 (38.5)	.78

^a^ In 2016, there were 119 individuals surveyed in the lockout group and 317 in the transitional medical assistance group, and in 2018, there were 59 and 394 individuals, respectively. Percentages are weighted to account for differential nonresponse.

^b^ Includes Asian and Native American.

In adjusted analyses, individuals who experienced a lockout were more likely to report being uninsured (33 [31.9%] vs 72 [18.7%]; *P* = .01) (Table 2). The 2 samples showed no significant differences in self-reports of having a usual source of care. However, those who experienced a lockout were less likely to report receiving needed medical care in the prior year (111 [64.9%] vs 561 [79.4%]; *P* = .001) and more likely to report that the quality of care received in the prior year was fair or poor (41 [21.4%] vs 61 [8.3%]; *P* = .001). Individuals who experienced a lockout were also more likely to report owing money for medical expenses (110 [63.5%] vs 210 [31.0%]; *P* < .001) and needing to borrow money, skip paying other bills, or pay other bills late to pay health care bills in last 12 months (66 [38.9%] vs 711 [20.9%]; *P* < .001). Self-reported health status or work-limiting disability status showed no significant differences.

**Table 2.** Access to Care and Health Status of Recent Medicaid Enrollees With Lockout Experience vs Those Without Lockout Experience

Characteristic	No. (Weighted %)		*P* Value
	Individuals With Recent Lockout Experience (n = 178)^a^	Individuals With No Recent Lockout Experience (n = 711)^a^	
Currently uninsured	33 (31.9)	72 (18.7)	.01
Has usual source of care other than urgent care or ED	113 (67.0)	512 (73.2)	.14
Needed medical care in last 12 mo and got it	111 (64.9)	561 (79.4)	.001
Quality of medical care received in the last 12 mo was fair or poor	41 (21.4)	61 (8.3)	.001
Currently owes money for medical expenses	110 (63.5)	210 (31.0)	<.001
Has had to borrow money, skip paying other bills, or pay other bills late to pay health care bills in last 12 mo	66 (38.9)	148 (20.9)	<.001
Self-reported physical and mental health			
Excellent or very good	65 (36.5)	310 (43.9)	.16
Good	68 (41.4)	258 (37.7)	.42
Fair or poor	35 (21.2)	130 (18.0)	.42
Missing	10 (0.5)	13 (0.4)	.92
A physical, mental, or emotional problem limits ability to work	25 (16.1)	91 (13.9)	.55

Abbreviation: ED, emergency department.

^a^ In 2016, there were 119 individuals surveyed in the lockout group and 317 in the transitional medical assistance group, and in 2018, there were 59 and 394 individuals, respectively. Estimates are adjusted for sex, age, and race/ethnicity. Percentages are weighted to account for differential nonresponse.

## Discussion

To our knowledge, this is the first study to compare recent Medicaid enrollees with and without lockout experience. Limitations included a cross-sectional study design that prevented causal inference, small sample sizes, and use of a single state where lockouts were relatively short-term (ie, at most 3 months). Although weighting was used to account for low response rates, our TMA sample overrepresented older, white enrollees (eTable 1 in the Supplement). Lockouts may disrupt access to care, which could account for some of the barriers identified in this study. Further research is needed to prospectively identify the effects of lockouts on access and long-term health outcomes.

## Notes

Supplement. **eTable 1.** Demographics of Survey Sample and Sampling Frame **eTable 2.** Demographics of TMA Enrollees and Parents/Caretakers in BadgerCare
