# Supplementary material for: Access to Care Among Individuals Who Experienced Medicaid Lockouts After Premium Nonpayment
Source: JAMA Netw Open. 2019 Nov 6;2(11):e1914561. doi: 10.1001/jamanetworkopen.2019.14561 (PMC6865263; doi:10.1001/jamanetworkopen.2019.14561)
Supplement: Supplement. — eTable 1. Demographics of Survey Sample and Sampling Frame eTable 2. Demographics of TMA Enrollees and Parents/Caretakers in BadgerCare [file jamanetwopen-2-e1914561-s001.pdf]

Supplementary Online Content

Saloner B, Dague L, Friedsam D, Voskuil K, Serna Borrero N, Burns M. Access to care among individuals who experienced Medicaid lockouts after premium nonpayment. *JAMA Netw Open*. 2019;2(11):e1914561. doi:10.1001/jamanetworkopen.2019.14561

eTable 1. Demographics of Survey Sample and Sampling Frame
eTable 2. Demographics of TMA Enrollees and Parents/Caretakers in BadgerCare

This supplementary material has been provided by the authors to give readers additional information about their work.

© 2019 Saloner B et al. *JAMA Network Open.*

eTable 1. Demographics of Survey Sample and Sampling Frame

		Survey	Sampling Frame
Male		23.30	26.17
Older than 35		61.87	52.95
Race/Ethnicity			
	White, Non-Hispanic	70.50	62.53
	Black, Non-Hispanic	9.95	17.40
	Spanish, Hispanic, or Latino	6.84	8.92
	Other race/Mixed race (Asian, Indian), not Hispanic	9.79	11.61
	Missing	2.86	NA

Notes: Authors' analysis of survey data collected in 2016 and 2018 of former enrollees in the Wisconsin Transitional Medical Assistance Medicaid program versus the Sampling frame.

© 2019 Saloner B et al. *JAMA Network Open.*

eTable 2. Demographics of TMA Enrollees and Parents/Caretakers in BadgerCare

		Parents/Caretakers in BadgerCare	TMA Enrollees	
Male		23.76	23.29	
Older than 35		70.88	61.88	***
Race/Ethnicity				
	White, Non-Hispanic	73.56	70.90	*
	Black, Non-Hispanic	8.73	9.99	
	Spanish, Hispanic, or Latino	6.92	6.87	
	Other race (Asian, Indian), not Hispanic	6.29	6.97	
	Mixed race, not Hispanic	3.66	2.88	
	Missing	0.84	2.39	***
High school diploma or greater than high school		93.62	79.07	***
Household annual income < $30,000		54.93	58.83	**
Household composition				
	Lives alone	8.40	6.92	*
	Lives with spouse	30.07	30.66	
	Lives with others	55.48	59.91	
	Missing	6.06	2.51	
Two or more household members below 19		42.15	38.46	**
Self-reported physical and mental health				
	Excellent, Very good	42.42	43.88	
	Good	39.56	37.68	
	Fair, Poor	17.95	18.04	
	Missing	0.07	0.425	
A physical, mental, or emotional problem limits ability to work at job		15.43	13.89	
Sample size		316	711	

Notes: Authors' analysis of survey data collected in 2016 and 2018 of former enrollees in the Wisconsin Transitional Medical Assistance Medicaid program who did not have experience with "lockouts" for non-payments of premiums versus Parents/Caretakers. *P<.1, **P<.05, ***P<.01

© 2019 Saloner B et al. *JAMA Network Open.*
